# Genome of *Raphanus sativus* L*.* Bakdal, an elite line of large cultivated Korean radish

**DOI:** 10.3389/fgene.2024.1328050

**Published:** 2024-01-18

**Authors:** Han Yong Park, Yu-jin Lim, Myunghee Jung, Subramaniyam Sathiyamoorthy, Seong Ho Heo, Byeongjun Park, Younhee Shin

**Affiliations:** ^1^ Department of Bioresource Engineering, Sejong University, Seoul, Republic of Korea; ^2^ Research and Development Center, Insilicogen Inc., Yongin-si, Gyeonggi-do, Republic of Korea; ^3^ Institute of Breeding Research, DASAN Co., Ltd., Pyeongtaek, Republic of Korea

**Keywords:** Korean radish, genetics, kimchi, breeding, *Raphanus sativus*

## 1 Introduction

Kimchi is a common and iconic food in Korean culture and is recognized globally as a healthy food (Surya and Nugroho, 2023). The process of kimchi preparation (kimjang) was included on the Intangible Cultural Heritage of Humanity list by UNESCO in 2013. The *per capita* consumption of kimchi in South Korea surpasses rice consumption (Cha et al., 2023). In Korean culture, fermented food plays a major role and traditional fermentation is one of the major food-processing techniques used to store vegetables during winter.

Kimchi is rich in antioxidants, vitamins, digestive enzymes, and minerals and has anti-cancer, anti-diabetes, and anti-inflammatory properties (Lee et al., 2016). Radish (*Raphanus sativus*) is used to prepare different forms of kimchi, such as *kkakdugi*-kimchi (the second-most consumed kimchi in Korea), *chonggak*-kimchi, and *dongchimi-*kimchi (Song et al., 2021; Surya and Nugroho, 2023). Radishes account for 10% of the total vegetable cultivation land in South Korea (https://kostat.go.kr). The burgeoning global popularity of Korean kimchi is increasing the demand for Korean radishes. The need for the sustainable cultivation of radish is growing. Countries like South Korea with small geographic areas are not able to increase the area devoted to agriculture. Instead, crop yield can be increased by establishing new plant traits with the aid of breeding science and technology.

Breeding in the genomic era has become more sophisticated with the development and refinement of technologies that include sequencing. The continuing trend of decreasing cost of genome sequencing and assembly has accelerated the adaptation of digital breeding methods to reveal genetic associations (Marks et al., 2021; Jeon et al., 2023). Vegetable crop varieties have been developed for resistance to various environmental stresses and to meet market demand phenotypes such as taste, aroma, and size. In conventional breeding practices, breeders select desirable traits randomly from nature through manual phenotype assessment. The selected variety is highly documented for a single phenotype, but the abstraction of biological characteristic spectra, such as biotic and abiotic stress resistance, by breeders is not simultaneous with variety/trait selection. Instead, breeders subject the selected varieties to various crossover events to develop new varieties according to the demand and supply in the seed market. This approach is tedious and time-consuming, and can be improved through rapid breeding techniques (Wanga et al., 2021), and genome selection (Budhlakoti et al., 2022). Specifically, breeding through genome selection is comparatively effective, whereas an individual crop has a pan-genome, which includes heterogeneous genomes. The decreased sequencing costs are encouraging plant breeding scientists to sequence single/target genomes to thousands of genomes aid to construct pan-genomes (Li et al., 2023).

Developing an economically important new radish variety is important in the Korean food industry to retain the aroma, pungency, and taste of kimchi. There are numerous varieties and traits of *R*. *sativus* and various breeding schemes worldwide aim to develop new traits for this species. The major obstacle in breeding schemes is retaining the crossbreed for multiple generations (i.e., the elite line for each trait/variety) (Kang et al., 2016; Epstein et al., 2023). There are hundreds of economic elite lines available globally; in South Korea, the term “Korean food” specifies that all raw materials used to prepare the food item originate in South Korea (Song et al., 2021). According to the Korean Seed Association, the demand for radish seeds is growing annually. In 2022, the 90.9 tons radish seeds that were produced sold for 445.7 billion Korean won. The chromosomal scale assembled genomes available for the radish crop (i.e., wild species adapted for cultivation that are included in the Raphanus pan-genome) are markedly fewer that the existing number of elite lines. In particular, the genome of the Korean radish cultivar *R*. *sativus* cv. WK10039 was recently decoded using a chromosome scale assembly (Cho et al., 2022). In this study, in addition to the WK10039 genome, we generated the chromosome-scaled genome assembly of *R*. *sativus* L. Bakdal, a widely cultivated radish line in South Korea which is exclusively used for breeding autumn and summer radishes at commercial breeding companies.

## 2 Values of the data

The Bakdal genome is an additional genetic resource for elite radish lines. Furthermore, the genome data will provide a basis and reference for more detailed studies of the genetic variation responsible for stable elite lines. Finally, the genome data may be a valuable resource for conducting comparative analyses of elite and wild varieties of the genus *Raphanus*, which could improve the genome selection process in molecular-assisted breeding.

## 3 Materials and methods

### 3.1 Sample collection for genomic DNA extraction

**FIGURE 1:**
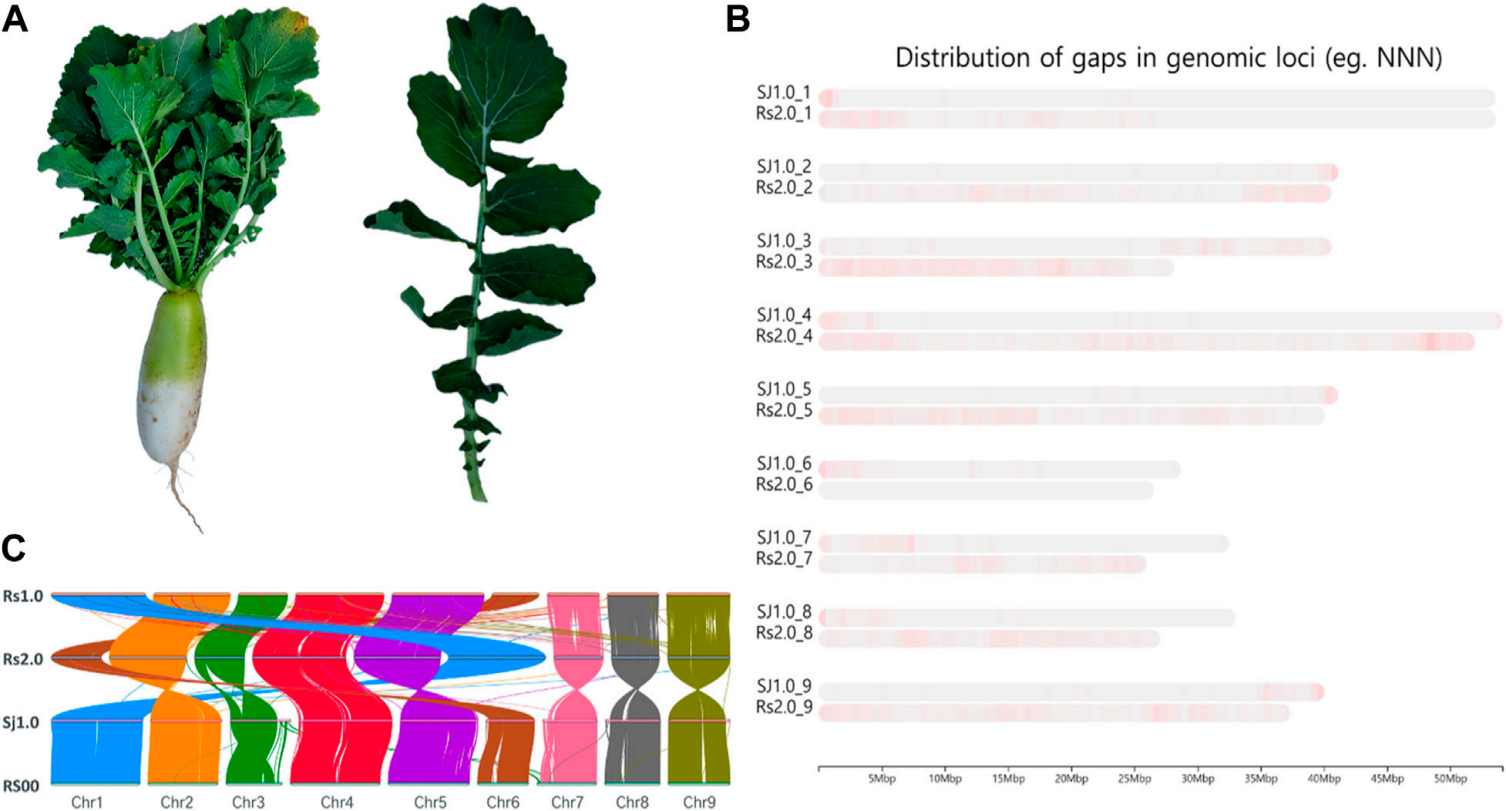
Summary of the sequencing. **(A)**. Phenotype illustration of the Bakdal radish variety used for sequencing in this article. **(B)**. Chromosome level comparison between *Raphanus sativus* cv. WK10039 (Rs), *R. sativus* var. Longipinnatus (RS), and Bakdal (Sj1.0), with improved gap assembled genome (green and violet color represent the gaps in chromosomes). **(C)**. Synteny representation of the Bakdal variety with previous versions of radish genomes.

*Raphanus sativus* L. Bakdal seeds were obtained from the Rural Development Administration, National Institute of Agricultural Science (https://genebank.rda.go.kr/). The seeds were maintained under accession number IT100672. Seeds were sown under natural polyhouse conditions established at Sejong University in June 2020. A photograph of the sample is shown in Figure 1A. Four-week-old plant leaves were collected for genome sequencing and immediately stored in liquid nitrogen.

### 3.2 DNA sequencing and *de novo* genome assembly

Total DNA was isolated from the samples individually according to standard sequencing protocols. DNA was prepared using the TruSeq Nano DNA Prep Kit for Illumina sequencing and the SMRTbell Express Template Preparation Kit (101-357-000; Pacific Bioscience, hereafter PacBio). Each isolated DNA was sequenced using two different sequencing methods: Sequel (PacBio) and Novaseq6000 (Illumina), which are established long- and short-read sequencing techniques. The experiment was performed using DNALink, an authorized service provider in South Korea. The Illumina paired-end and Hi-C sequences were initially subjected to filtering of technical artifacts (i.e., base calling error, Phred quality score [Q] ≤20)), and adapters using the Trimmomatic-v0.32 method (Bolger et al., 2014). The PacBio reads were error corrected. The corrected reads were used for the initial *de novo* draft version of the Radish genome with FALCON v1.8.1 haplotype assembler (Chin et al., 2016) and assembly polished with Pilon (Walker et al., 2014). Scaffold artifacts were corrected with 30 GB of Hi-C genome sequence using the HiRise (Putnam et al., 2016) method. Finally, the assembled contigs were scaffolded to the chromosomal scale using the reference radish cultivar, *R*. *sativus* cv. WK10039 using the RagTag v2.1.0 method (Alonge et al., 2022). The scaffolded contigs were assessed for completeness using BUSCO v5.4.7, using the Viridiplantae_odb10 reference dataset (Seppey et al., 2019).

### 3.3 De contamination and estimation of genome size

Initially, reference databases were prepared in two sequential steps: bacterial contamination and organellar genomes. First, the complete bacterial draft and reference genomes from GenBank were used as references, and the complete organelle genomes (i.e., mitochondria and plastids) were used as references to remove reads from the raw sequence reads. All reference mapping of preprocessed reads was performed using Bowtie2 v2.2.8 (Langmead and Salzberg, 2012). All Illumina preprocessed sequences from the paired-end library were subjected to genome size estimation using the *k*-mer based method previously used for the panda genome (Li et al., 2010). The *k*-mer frequencies (*k*-mer size = 17) were obtained using Jellyfish v2.1.3 (Marçais and Kingsford, 2011). The genome coverage depth was calculated as (*k*-mer Coverage Depth × Average Read Length)/(Average Read Length–*k*-mer size +1). The genome size was calculated as: Total Base Number/Genome Coverage Depth. The *k*-mer Coverage Depth was the major peak in *k*-mer distribution.

### 3.4 Prediction and classification of repeat regions

Repeat regions in the *R*. *sativus* L. Bakdal genome were predicted using RepeatModeler (www.repeatmasker.org/RepeatModeler/) and classified into subclasses using the reference Repbase v20.08 database (www.girinst.org/repbase/) (Bao et al., 2015). Finally, the repeats were masked in the genome using RepeatMasker v4.0.5 (www.repeatmasker.org) with RMBlastn v2.2.27+. The results are shown in Supplementary Table S2.

### 3.5 Gene prediction and annotation

Genes from the *R*. *sativus* L. Bakdal genome were predicted using an in-house gene prediction pipeline that included an evidence-based gene modeler (EVM), *ab initio* gene modeler, and a consensus gene modeler. The *ab initio* gene modeler and EVM, including exonerate (Slater and Birney, 2005), and AUGUSTUS (Stanke et al., 2006), were trained with several genomes (Figure 1B). The final gene and transcript models were optimized with a consensus gene modeler with EVidenceModeler (Haas et al., 2008) and annotated with Trinotate v.3.0.1 (Bryant et al., 2017).

### 3.6 Preliminary analysis

Initially, the size of the *R. sativus* L. Bakdal genome was estimated to be 427.71 (Supplementary Figure S1), based on ∼23 GB of short-read Illumina sequences (Table 1A). A 57 GB error corrected long-read sequence was assembled into 398.6 Mb a 40 MB N50 size gap-free chromosomal scale assembly as illustrated in Supplementary Figure S2 and Supplementary Tables S2, S5. In total, 199 MB (50.03%) assembled genomes were filled with repeat regions, and approximately 30% of the repeats were unclassified into known subclasses of the repeat elements (Supplementary Table S1). The completeness score of the assembled BUSCO genome was 99.9% (Supplementary Figure S3). Furthermore, 50,281 genes were predicted to have an average length of 2,471 bases with other *R. sativus* genomes (Table 1B, Supplementary Figure S4, Supplementary Tables S3–S5). Finally, 68.64% of the genes had homologous sequences in the SwissProt database, and 62.24% of the genes were mapped to the Kyoto Encyclopedia of Genes and Genomes (KEGG) pathway database. Moreover, 65.44% of the genes had an identified gene ontology (GO). The genic region of the genome was annotated well using reference databases (Table 1C). The Bakdal-assembled genome was mapped to *R*. *sativus* cv. WK10039 (RS.2.0) (Figure 1B) using the RagTag method to improve scaffold assembly at the chromosome scale. In this study, we generated the genome of the Bakdal line, a commercially important crop widely cultivated in Korea for kimchi production. Compared with RS2.0, the novel genome has additional bases on chromosome three by synteny assessment (Figure 1B). Finally, chromosome one and six were interchanged from previous assemblies (Figure 1C). With the use of this genome research, a full analysis of the genetic information of Bakdal radish has been shown. The most recent discoveries will be of great assistance in the process of incorporating genetic modification into radish cultivars that are predominantly farmed for commercial reasons.

**TABLE 1:** Summary of the sequencing to annotation of the Bakdal chromosomal genome assembly (Sj1.0) along with *Raphanus sativus* cv. WK10039 (Rs2.0).

A. Sequencing and assembly summary		
Descriptions	SJ1.0	Rs2.0
PacBio	57 GB	
Illumina short reads	23 GB	
Illumina Hi-C	30 GB	
Number of Scaffolds	24,155	6,424.00
Total length	398,011,222	433,430,583
N50	40,361,493	37,282,294
Average length	16,477.38	67,534.62
Minimum length	200	698
Maximum length	54,127,229	53,709,999
Number of N	746.468 (0.19%)	269,710 (0.06%)
BUSCO [brassicales_odb10]	C:98.7%[S:90.9%,D:7.8%], F:0.4%, M:0.9%	C:98.6%[S:77.7%,D:20.9%], F:0.3%, M:1.1%
Number of Chromosomes	9	9
Number of Unplaced	24,146	6,413
B. Gene prediction summary		
Number of genes	50,281	56,517
Average gene length (bp)	2,471.04	2,437.05
Gene coverage (%)	31.22	31.78
GC in CDS (%)	46.31	45.99
Exon:Gene	5.32	6.24
Average exon length (bp)	220.16	236.25
Exon coverage (%)	14.8	19.21
Average intron length (bp)	300.58	183.95
Intron coverage (%)	16.41	12.56
Number of rRNAs	966	1,081
Number of tRNAs	1,449	855
C. Functional Annotation Summary		
Database	No. ORFs (%)	
SwissProt	34,514 (68.64)	
KEGG	31,297 (62.24)	
eggong	354 (0.70)	
Pfam	33,663 (66.95)	
GO	32,902 (65.44)	
No hit	11,718 (23.31)	

## Data Availability

The original contributions presented in the study are publicly available. This data can be found here: Sequence Read Archive repository under accession number PRJNA1026765/(10.6084/m9.figshare.24313855).
