# Supplementary material for: Genome of Raphanus sativus L. Bakdal, an elite line of large cultivated Korean radish
Source: Front Genet. 2024 Jan 18;15:1328050. doi: 10.3389/fgene.2024.1328050 (PMC10831357; doi:10.3389/fgene.2024.1328050)
Supplement: Supplementary file 1 [file Presentation1.PPTX]

## Slide 1
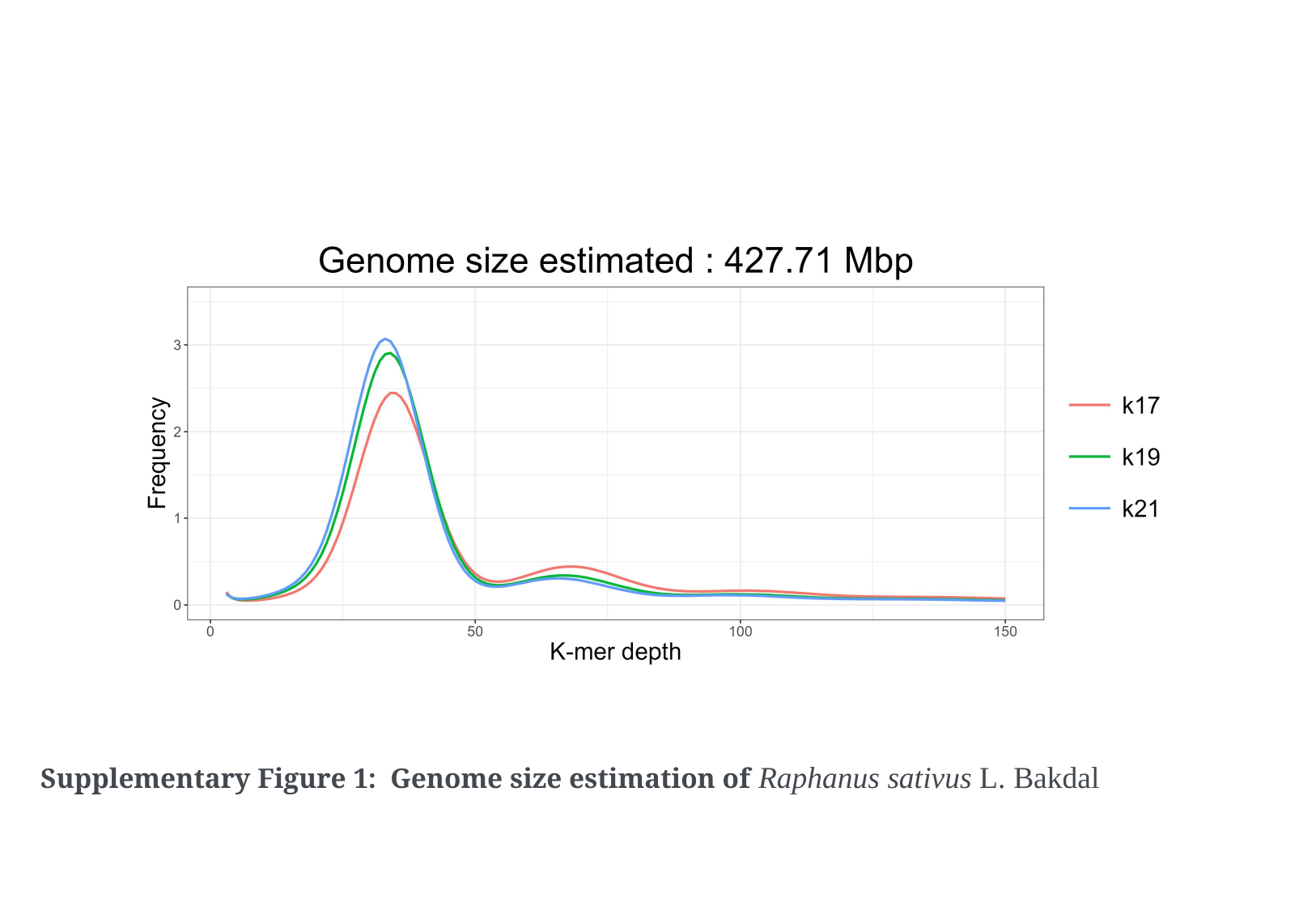

Supplementary Figure 1: Genome size estimation of Raphanus sativus L. Bakdal

## Slide 2
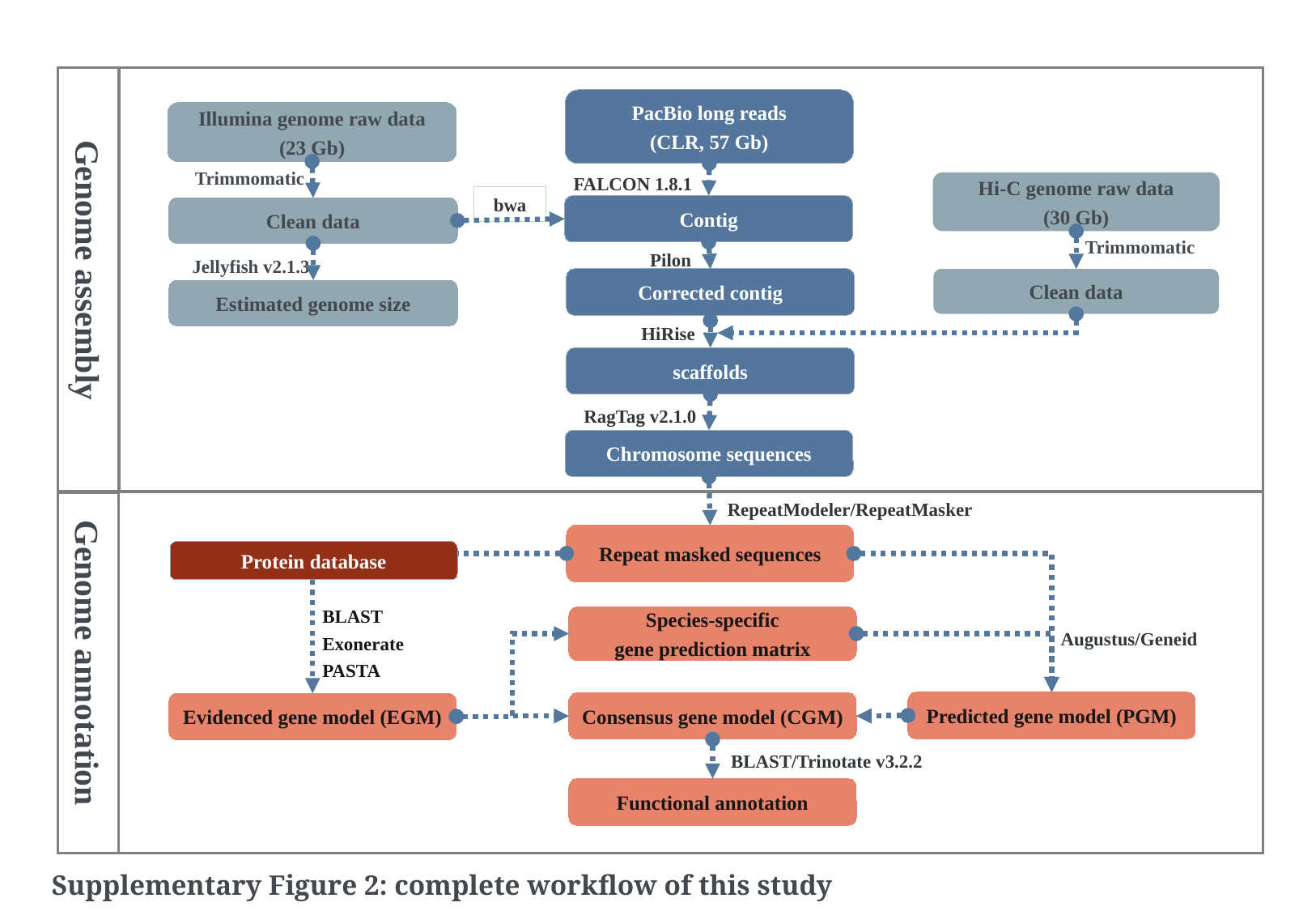

PacBio long reads
(CLR, 57 Gb)
Illumina genome raw data
(23 Gb)
FALCON 1.8.1
Trimmomatic
Hi-C genome raw data
(30 Gb)
bwa
Contig
Clean data
Trimmomatic
Pilon
Jellyfish v2.1.3
Corrected contig
Clean data
Estimated genome size
Genome assembly
HiRise
scaffolds
RagTag v2.1.0
Chromosome sequences
RepeatModeler/RepeatMasker
Repeat masked sequences
Protein database
Species-specific
gene prediction matrix
BLAST
Exonerate
PASTA
Augustus/Geneid
Genome annotation
Predicted gene model (PGM)
Consensus gene model (CGM)
Evidenced gene model (EGM)
BLAST/Trinotate v3.2.2
Functional annotation
Supplementary Figure 2: complete workflow of this study

## Slide 3
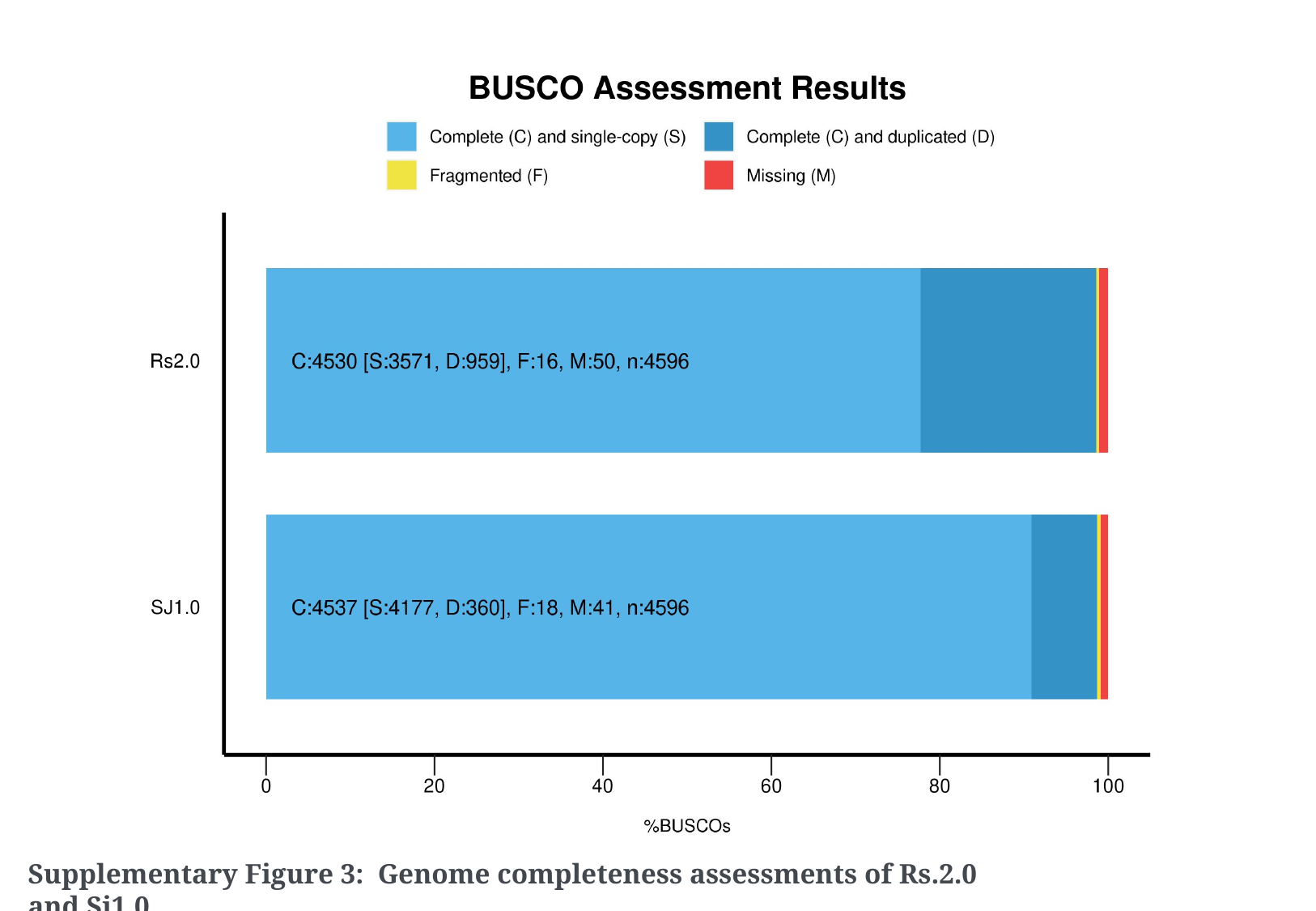

Supplementary Figure 3: Genome completeness assessments of Rs.2.0 and Sj1.0

## Slide 4
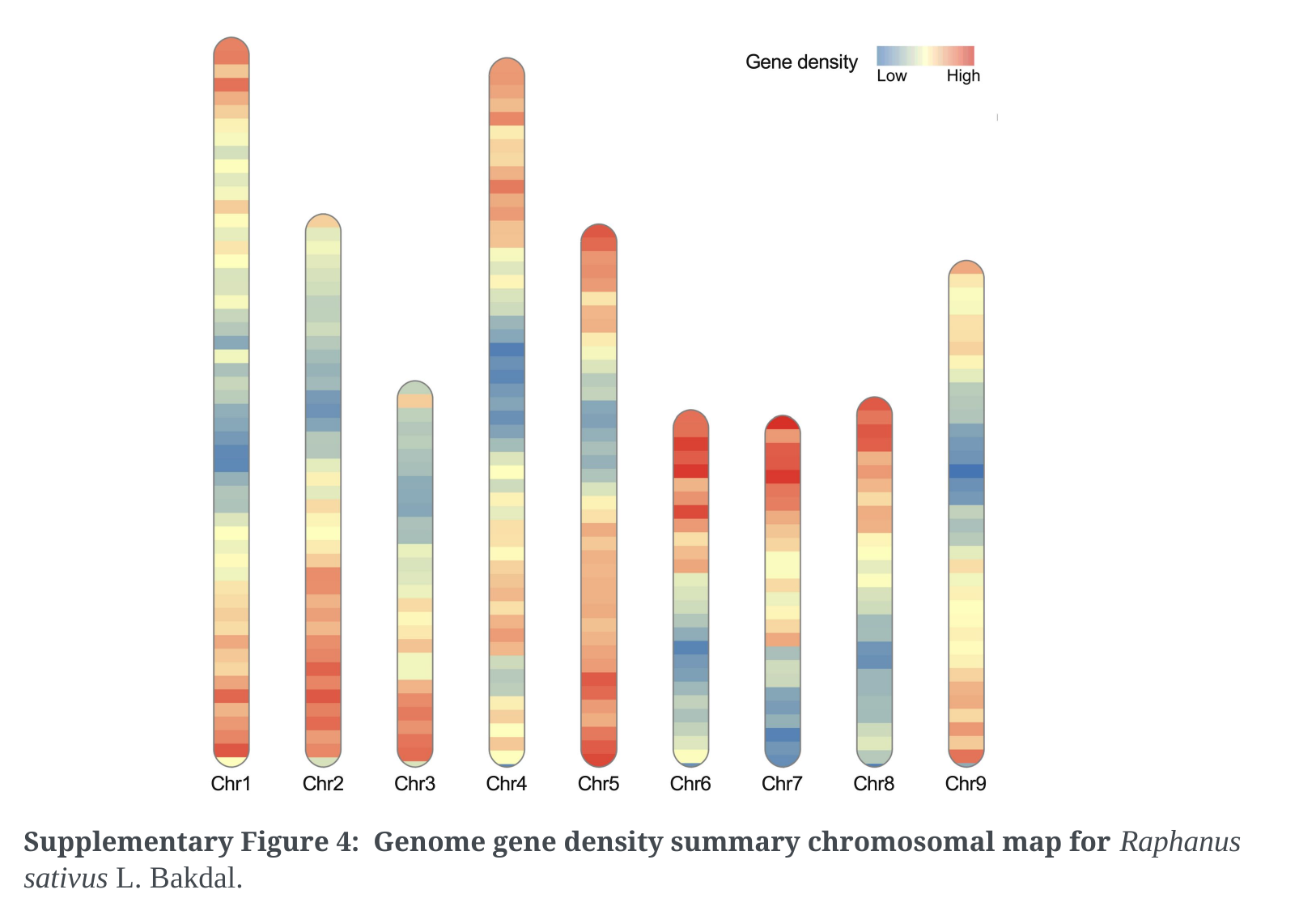

Supplementary Figure 4: Genome gene density summary chromosomal map for Raphanus sativus L. Bakdal.
